# Mapping the Evolution of Mercury (Hg) Research in the Amazon (1991–2017): A Scientometric Analysis

**DOI:** 10.3390/ijerph16071111

**Published:** 2019-03-28

**Authors:** Lilian de C. Moraes Pinto, José G. Dórea, José Vicente Elias Bernardi, Leonardo Fernandes Gomes

**Affiliations:** 1 Programa de Pós-Graduação em Ciências Ambientais, Faculdade UnB Planaltina, Planaltina, Distrito Federal 73345-010, Brazil; bernardi@unb.br; 2 Faculdade de Ciências da Saúde, Universidade de Brasília, Asa Norte, Brasília, Distrito Federal 70919-970, Brazil; jg.dorea@gmail.com; 3 Núcleo de Estudos e Pesquisas Ambientais e Limnológicas—NEPAL, Faculdade UnB de Planaltina, Planaltina, Distrito Federal 73345-010, Brazil; leof.ciamb@gmail.com

**Keywords:** scientometry, tropical rain forest, methylmercury, co-citation analysis, co-authorship analysis

## Abstract

Because the Amazon rain forest is ecologically relevant on a global scale, we applied scientometric techniques to integrate studies dealing with mercury research in this unique ecosystem between 1991 and 2017. Using a combination of co-authorship and co-citation analyses, keyword mapping and overlay visualization of topics in the field, this article identified three major areas in the 26-year period of mercury research: (1) human exposure to mercury (artisanal small-scale gold mining-ASGM) and methylmercury through fish consumption, and their respective risks for human health; (2) mercury accumulation in the environment and its relation to ASGM and atmospheric concentration; and (3) mercury geochemistry and its presence in soils, sediments, and water. The paper also identified the leading institutions related to the published research and respective influential scholars in the context of this study. Overall, the analyses revealed patterns of convergence and divergence between authors, specialization, and interdisciplinary engagement in mercury investigation, thus highlighting strengths and weaknesses of research topics in the field. This scientometric approach could be a useful tool to monitor/assess the implementation of the Minamata Convention.

## 1. Introduction

Mercury (Hg) is hazardous to human health and wild life. Because of its high toxicity and capacity to accumulate in food webs, Hg is classified as a persistent toxic substance (PTS). Hg occurs from natural sources, although it also enters the environment from anthropogenic sources [1]. Its methylated form, methylmercury (CH_3_Hg), is considered the most toxic of the organometallic compounds [2], and it is easily absorbed by organisms, as it is able to bioaccumulate within an individual over time and biomagnify through the food chain [3,4]. Although the neurotoxic consequences of mercury are well-established [5], recent studies show that adverse health effects can include behavioral, developmental, endocrinological, immunological, nephrological, and reproductive outcomes [6].

The implications of environmental mercury contamination are well-documented [7] and have led to the need for international cooperation regarding its control. The Minamata Convention on Mercury is a legally binding international agreement designed to control, reduce, or eliminate major anthropic sources of Hg [4]. Its ultimate goal is to protect human health and the environment [8]. The driving force of most environmental Hg research is the concern with human exposure to methyl-Hg [7]. Humans can be exposed to this compound through consumption of contaminated fish [9,10]. Occupational activities, especially for those who deal with gold amalgamation, constitute the main route of exposure to inorganic Hg [11]. 

The complex and diverse Amazon rain forest has been a matter of public concern for decades. Worldwide attention focuses on this unique ecosystem regarding environmental mercury issues with both regional and global ecological relevance [12,13]. Hydroelectrical projects, expansion of agricultural frontiers for exportable commodities, and artisanal gold mining have left a trail of environmental destruction in the Amazon. Although all of these environmentally impacting activities have direct and indirect influences on the mass balance of available mercury, it is undoubtedly artisanal gold mining activity that has received the most attention. In the Amazon rain forest, fine gold is amalgamated with metallic mercury, thus, polluting the environment, either directly into rivers or into the atmosphere [14,15,16]. 

In fact, due to regional and worldwide interest, mercury in the Amazon region has been studied with several different foci, such as environmental sciences [17], ecotoxicology [18], occupational health, and public health [19]. Therefore, the aim of this study was to make a scientometric analysis of mercury research in the Amazon in order to identify and integrate published Hg research related to the fragile and threatened Amazonian ecosystem.

Thus, we identified (i) the published mercury-related research in the Amazon; (ii) the most significant authors in the area and how co-authorship networks have been established; (iii) and how publications and authors were grouped according to different research topics.

## 2. Materials and Methods

We searched the database of the Web of Science (WoS) Core Collection for mercury-related publications in the Amazon. An advanced search was performed using the following terms: TI (Title) = (*mercur* OR hg) AND TS (Topic) = (amazon*). The search was restricted to the years between 1991 and 2017, to articles in English, and to publications listed through 20 June 2018. Among other internationally known databases (e.g., Scopus, Google Scholar, and PubMed), the WoS database is extensively used for scientific research retrieval [20]. It includes over 13,900 journals that are considered high quality and have a strong impact. It is a rich dataset used across multiple academic fields [21]. The year 1991 is when author abstracts and keywords started becoming available on the WoS platform.

The information about the authors, years of publication, and institutions was extracted in text format and evaluated with the HistCite™ software, version 9.8.24 (Philadelphia, PA, USA). To evaluate if the number of publications presented an increasing tendency over the years, we performed a linear regression through the lm function, a vegan package of the R statistical software (R Foundation for Statistical Computing, Vienna, Austria).

We used the VOSViewer™ software, version 1.6.8 (Leiden, the Netherlands), which builds network connections of scientific publications, scientific journals, researchers, research organizations, countries, keywords (or terms based on co-authorship), co-occurrence, citation, and bibliographic coupling (or co-citation links). It counts both the number of links and the total strength of those links to plot the graphical representation, in which the size of a circle represents relevance of a topic while network connections show the link strength of that topic [22].

In order to obtain the networks of interactions between the main authors that published on mercury in the Amazon region, a network of co-authors was created. The selected authors were those who participated in at least five publications. Each node corresponded to a co-author, while the proportion of node sizes matched the number of publications by each author. To evaluate the main approaches of the publications, the title and abstract words (keywords) were also extracted and analyzed. For a word to be selected, it should have occurred in at least ten different publications. Keywords were selected as node types, and the dimensions of the nodes correlated to the number of records of each word. Additionally, we assessed the main references cited in the publications to get a more comprehensive interpretation of the evolution of the field. Therefore, we established that for a reference to be included in the network, it should have been cited in at least 20 publications. In the same way, the citations were grouped and the dimensions of the rectangles referred to the number of citations of these publications. 

Analyses performed on databases often requires filtering, corrections, and normalizations; these procedures are prone to failures, mainly due to errors in spelling, incoherence, and the occurrence of homonyms (that is, different authors with the same name). If an author’s name was presented in different forms in distinct articles, each name would be regarded as a separate author. This problem could greatly influence the accuracy of the results, and some authors could be under- or overrepresented. Thus, some modifications were necessary, and we manually applied standardization and normalization in the references. 

In relation to the dataset, this study was constrained by the fact that only the first cited author of a reference is recorded in the WoS database; therefore, the co-citation analysis was conducted using the first author exclusively, undervaluing the influence of the co-authors. Moreover, some journals list authors alphabetically, while others list authors by their contributions. In order to avoid the “alphabet bias” we double-checked the data used; none of the papers included their authors alphabetically. Therefore, there is no “alphabet bias” in this paper.

## 3. Results

**Figure 1:**
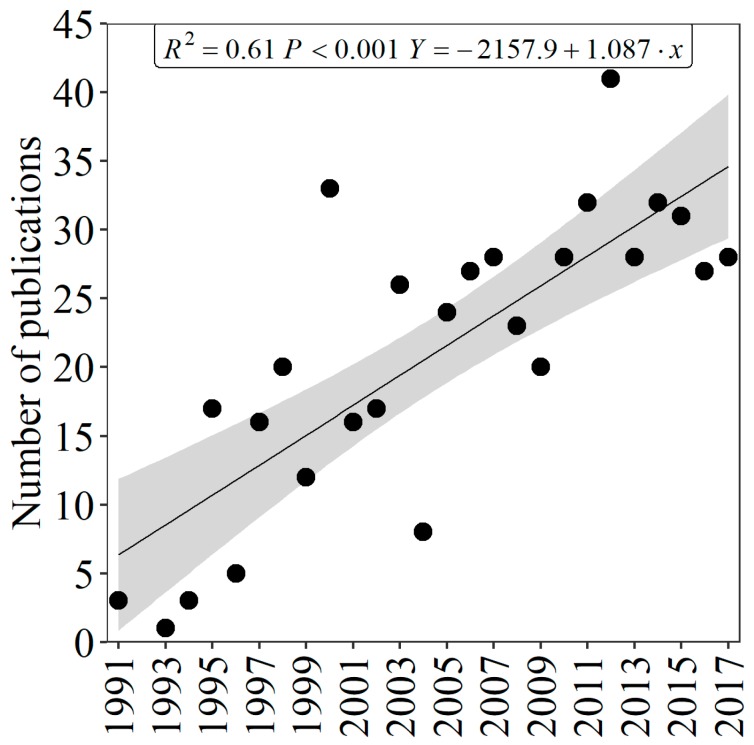
Number of publications about mercury in the Amazon between the years 1991 and 2017.

A total of 546 articles dealing with mercury in the Amazon were published in international peer reviewed journals (WoS). A summary of the number of publications over the years is shown in Figure 1; the year 2012 had the largest number of articles. 

**Figure 2:**
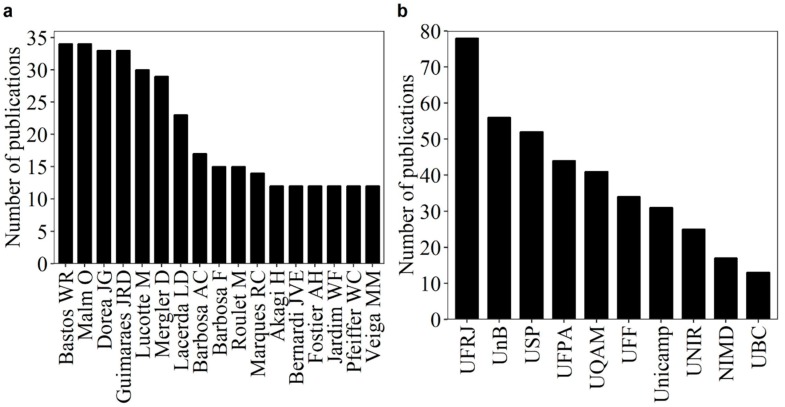
(**a**) Main authors and (**b**) institutions that most published about mercury in the Amazon between the years 1991 and 2017. Abbreviations: Universidade Federal do Rio de Janeiro (UFRJ); Universidade de Brasília (UnB); Universidade de São Paulo (USP); Universidade Federal do Pará (UFPA); Université du Québec à Montréal (UQAM), Canada; Universidade Federal Fluminense (UFF); Universidade de Campinas (Unicamp); Universidade Federal de Rondônia (UNIR); the National Institute for Minamata Disease (NIMD), Japan; and The University of British Columbia (UBC), Canada.

The first seven authors who published most on mercury in the Amazon have more than 20 publications (Figure 2a). Mercury in the Amazon has been studied in Brazilian institutions as well as by international groups in the Université du Québec à Montréal (UQAM), the National Institute for Minamata Disease (NIMD), and The University of British Columbia (UBC), which were among the ten that published the most on the subject (Figure 2b).

### 3.1. Co-authorship Analysis

**Figure 3:**
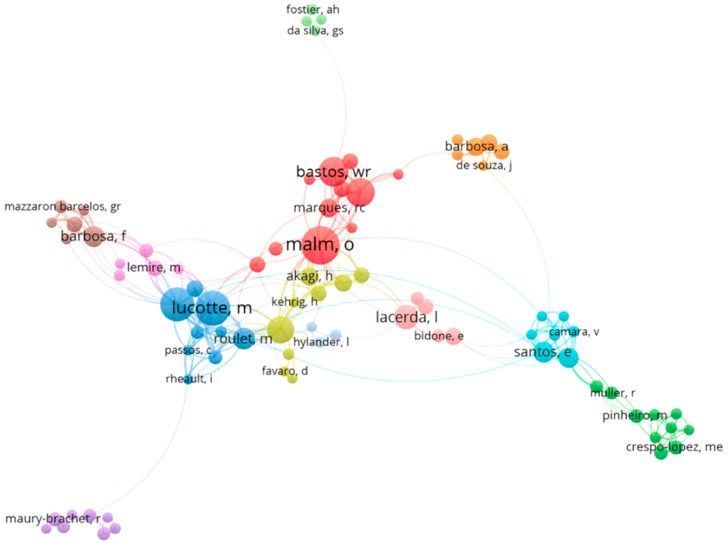
Co-authorship network regarding Hg research in the Amazon between 1991 and 2017.

Among the 546 papers analyzed, we identified 75 authors that qualified as nodes of the collaboration networks. These nodes made up 12 main collaborative clusters shown in Figure 3, which is complemented by information displayed in Table 1.

### 3.2. Keyword Analysis

**Figure 4:**
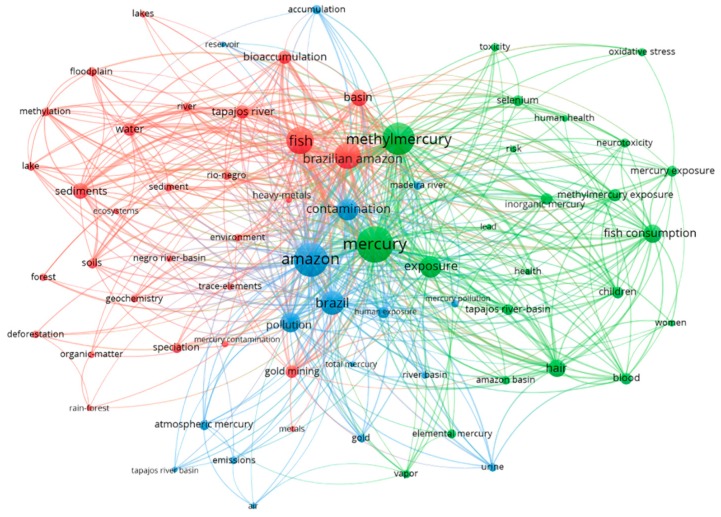
Networks of associations among the most used words in the titles and abstracts of publications on mercury in the Amazon between 1991 and 2017. The colors represent the clustering between them.

There were 2089 words listed by authors in the title and abstract from analyzed articles. After applying the criteria that the word should appear in at least ten publications, 93 words were identified, with 1419 links. After filtering to remove synonymous words and terms unrelated to the subject, we ended up with 69 words grouped in three main topic clusters (Figure 4). The main keywords ranked by frequency in papers are listed in Table 2.

### 3.3. Co-citation Analysis

**Figure A1:**
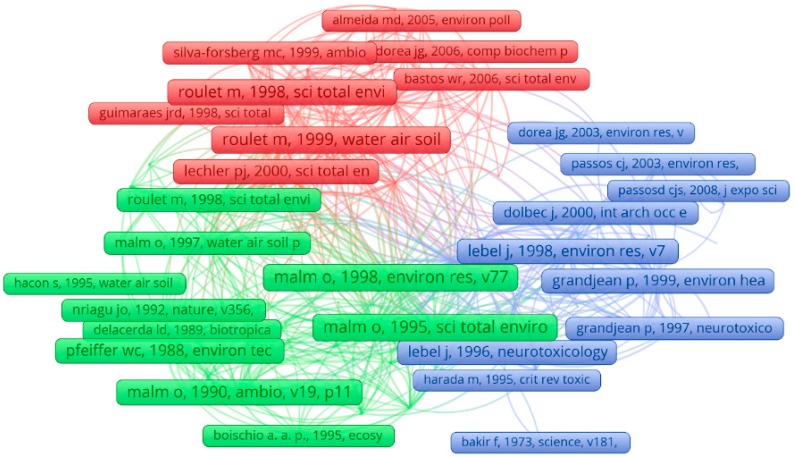
Reference networks about mercury research in the Amazon between 1991 and 2017. The colors represent the clustering between them.

The identification of the most cited publications revealed the leading authors and the key issues of interest in Hg research in the Amazon. It is important to clarify that the 12,811 references analyzed from the 546 articles in the database resulted in this list below (Table 3), indicating that the articles presented in it are the most cited within the papers that compose the database considered for this study (Appendix A; Appendix Figure A1). 

## 4. Discussion

Gold mining activities in the Amazon have left a trail of Hg contamination with consequences in ecotoxicology and human health. The scientometric analysis of Hg research in the Amazon identified scientific networks revealing topics of publications and research groups involved. Additionally, it also highlighted the topics of most interest. Although this approach to Hg ecotoxicology is new to the Amazonian ecosystem, this research tool has been successfully used in other environmental areas of study, such as low-carbon development [23], waste management [24], transportation [25], and ecological environments [26]. In keeping with the Minamata Convention recommendations, this work adds research-based data and information on environmental Hg, generated in the unique Amazon ecosystem.

### 4.1. Co-Authorship Analysis

Co-authorship is considered one of the most tangible indicators of research collaboration and accurately assists in tracking aspects of scientific networks. In a collaborative network it is common for a particular author to publish more than one paper with another particular author; opportunity and preferences play a role in increasing collaboration, favoring some interactions more than others [27].

The exhaustive work that goes into conducting studies concerning mercury contamination in the Amazon revealed an intricate co-authorship network. Each cluster in Figure 3 was due to the authors having published papers together, and clusters were connected because these authors often worked with colleagues outside their inner group. An author’s centrality or node size showed his/her relative relevance (e.g., Malm, O., Lucotte, M., Mergler, D. and Bastos, W.); the cluster position indicated closeness of association between groups. For instance, the cluster in gray (cluster 05; Table 1) was located at some distance from the others, and its position could be explained by resident French authors or by their unique focus on the part of the Amazonian basin that is found in French Guiana. Physical proximity might promote extensive collaboration in some clusters (e.g., cluster 02; Table 1); however, this network contained authors and institutions from several Brazilian states and nationalities, interacting within their cluster and with others, regardless of geographical limitations.

Table 1 shows that some authors can be seen in more than one cluster, as was the case of Guimarães, J.R.D., Bastos, W. and Dórea, J. Additionally, it indicated which clusters were more productive and which no longer worked in co-authorship. Because it is impossible for a single researcher to master several lines of endeavor in this broad field of ecotoxicology, it is likely that the enhanced scientific specialization among scientists has led to an increase in collaboration among authors with common interests, thus fostering the swift development and growing complexity of networks. Therefore, these data suggest a considerably collaborative framework, with distinct core research areas.

### 4.2. Keyword Analysis

The analysis of main keywords present in papers regarding Hg research in the Amazon (Figure 4) revealed three clusters, with different foci. 

The largest cluster (*n* = 29) focused on mercury in the environment and its relation to gold mining, dissolved organic matter, and other heavy metals, and was also related to mercury speciation and bioaccumulation in aquatic ecosystems. Frequently used terms included Brazilian amazon, fish, basin, sediments, water, soil, gold mining, and bioaccumulation.

The second largest cluster (*n* = 23) targeted human exposure to mercury and methylmercury through fish consumption, especially women and children, and its neurotoxic effects and risks to human health. Terms such as mercury, methylmercury, exposure, fish consumption, hair, and blood were common.

The third cluster (*n* = 17) included mercury geochemistry, pollution of river basins and reservoir contamination, as well as its association with atmospheric concentration. Terms that linked papers in this cluster encompassed Amazon, Brazil, contamination, pollution, atmospheric mercury, and accumulation.

### 4.3. Co-Citation Analysis

Citation analysis helps to understand how knowledge dynamics in Hg research in the Amazon are generated and interconnected through the years. The most cited references highlight the importance of the work of each research team; additionally, it paves the way for more research while influencing current knowledge [28]. 

Table 3 shows the ten most cited publications, thus emphasizing the relevance of the researched topic and the influential role of the respective research teams. Among these, Malm drew attention, with three works of his own (Malm 1990, 1995, and 1998) among the ten most cited. Another highlight was the author Lebel, who published two papers that appeared amongst the most cited (Lebel 1997, 1998). 

Results showed that three major clusters of author co-citations emerged (Figure A1): mercury in fish and humans in areas of gold extraction, geochemistry of soils and water bodies in the Amazon and its relationship with the presence of mercury, and potential effects of mercury contamination on human health. In these three major areas were the publications that have been mentioned the most by peers, regarding the presence of mercury in the Amazon.

In the period studied, the analysis of co-citations showed the main areas of research that contributed to the formation of knowledge in Hg contamination in the Amazon.

### 4.4. Main Research Limitations and Strengths

Inevitably, the results of this study are limited by certain features that need to be acknowledged. The reasons authors make specific citations may vary, as some authors cite peers, not because of the content they publish, but as a mutually friendly way to increase the count of citations or as a way to meet a requirement of the journal to which it is to be submitted [28]. Since it is impossible to separate behavioral citations such as those mentioned above, it is important to note that these instances may affect the study results. However, the number of citations motivated by some factor other than actual influence is considered a small percentage. 

Additionally, we focused on one document type, articles, thus excluding opinion pieces and letters to the editor, which may also add substantive references to WoS [21]. We analyzed only English language scholarly material, disregarding material originally published in other languages. It is important to mention that this study does not reflect a comprehensive account of Brazilian research into Hg in the Amazon, as we did not include papers from other databases, only the Web of Science Core Collection.

Finally, the data collection was carried out covering all articles within the indicated parameters until December 2017, which meant that the very most recent articles published certainly did not have enough time to appear in this study or be cited and appear in the networks of citations and co-citations. We are convinced that these limitations are justifiable, considering the scope of this study, but also feel that future research may benefit from expanding this work to include some of the remarks listed above.

As a strength, the paper demonstrates that scientometry can be used to evaluate/monitor the effectiveness of the Minamata Convention. This scientometric approach revealed information concerning mercury in a defined ecosystem, based on knowledge generated by scientists and experts. Thus, it might be useful regionally or globally for the periodic assessment of the implementation of the Minamata Convention.

## 5. Conclusions

This comprehensive scientometric evaluation of mercury research in the Amazon from 1991 to 2017 resulted in the following findings. Firstly, annual publications concerning mercury in the Amazon presented an increasing trend over the years, and Brazilian public universities were identified as being the top four institutions that published most about mercury in the Amazon. Secondly, we identified the top influential scholars in the context of this study: Bastos, Malm, Dórea, Guimarães, Lacerda, Lucotte, and Mergler. Our research revealed that authors formed twelve different clusters, with different research directions and strengths. Finally, analysis of the cited reference cluster provided a dynamic view of hot research topics during the period considered, corroborated by the keyword analysis results. These topics were: (1) human exposure to mercury (artisanal small-scale gold mining-ASGM) and methyl-Hg through fish consumption, and their respective risks for human health; (2) mercury accumulation in the environment and its relation to ASGM and atmospheric concentration; and (3) mercury geochemistry and its presence in soils, sediments, and water. 

Overall, this scientometrics study integrates scientific interest in mercury publications in the Amazon and helps to identify strengths and weaknesses of research areas, thus, adding guidance for future research.

## Figures and Tables

**Table 1:** Most common authors on Hg in twelve clusters in the Amazon co-authorship network, inferred from 546 publications from 1991 through 2017.

No.	Cluster Members (^1^)	Core Research Areas	Period
01	Malm, O. (34), Bastos, W. (24), Dórea, J. (22), Bernardi, J. (10), Diez, S. (8), Barbosa, A. (5), Fonseca, M. (5), Forsberg, B. (6), Guimarães, J. (9), Lacerda, L. (9), and Marques, R. (12).	Gold mining as a source of mercury exposure; spatial-temporal dynamics and sources of total Hg; neurological effects of Hg contamination in children; Hg bioaccumulation and biomagnification.	1991–2017
02	Pinheiro, M. (13), Crespo-Lopez, M. (8), Nascimento, J. (6), Herculano, A. (8), Muller, R. (7), Sarkis, J. (7), Silveira, L. (6), Oikawa, T. (6), and Vieira, J. (6).	Genotoxicity and health effects in humans exposed to Hg, especially women and children from riverside communities.	2000–2017
03	Lucotte, M. (30), Mergler, D. (29), Amorim, M. (8), Farella, N. (7), Lebel, J. (6), Passos, C. (5), Rheault, I. (5), and Roulet, M. (15).	Geochemistry of mercury in soils, sediments, and water; neurotoxic effects of low-level methylmercury contamination; the relation between fish consumption and human contamination; and the way anthropogenic factors influence mercury dynamics.	1996–2017
04	Guimarães, J. (21), Bastos, W. (9), Akagi, H. (12), Pfeiffer, W. (11), Favaro, D. (5), Fostier, A. (6), Kehrig, H. (5), Vasconcellos, M. (5), and Branches, F. (9).	Hg pollution in gold mining areas and manmade reservoirs, concerning mainly mercury levels in riverine and indigenous populations.	1991–2006
05	Boudou, A. (7), Bourdineaud, J. (5), Charlet, L. (5), Cossa, D. (7), Grimaldi, M. (7), Guedron, S. (6), Maury-Brachet, R. (8), and Richard, S. (5).	Hg in aquatic environments and the effects from gold mining in fish and human contamination, with a unique focus on the part of the Amazonian basin that is found in French Guiana.	2001–2014
06	Cleary, D. (5), Brabo, E. (13), Camara, V. (6), de Jesus, I. (5), Loureiro, E. (5), Mascarenhas, A. (6), and Santos, E. (14).	Hg contamination in fish and riverside communities, especially in children.	2000–2015
07	Barbosa, F. (14), Grotto, D. (9), Garcia, S. (5) Valentini, J. (5), Braga, G. (5), and Barcelos, G. (7)	Molecular behavior of mercury and its genetic effects in humans.	2009–2015
08	Barbosa, A. (12), Dórea, J. (10), Boischio, A. (6), Jardim, W. (9), de Souza, J. (5), and Ferrari, I. (6).	Hg contamination in fish and in hair of different populations; fish consumption and nutritional status; mercury exposure and serum antinuclear antibody; cardiovascular risks of Hg contamination; Hg bioaccumulation and biomagnification.	1995–2010
09	Fillion, M. (8), Lemire, M. (9), Mertens, F. (5), Nyland, J. (6), and Silbergeld, E. (6).	Hg molecular behavior; Hg contamination in a fish-eating population, neurotoxic sequelae; mercury and selenium concentration patterns.	2007–2017
10	Lacerda, L. (18), Bidone, E. (7), Campos, R. (7), Castilhos, Z. (11), and Hacon, S. (9).	Ichthyofauna and human exposure to mercury through fish consumption.	1991–2004
11	Jardim, W. (5), Fadini, P. (5), Fostier, A. (6), and da Silva, G. (5).	Mercury chemistry, including the part played by dissolved organic matter in the mercury cycle, gaseous mercury and mercury in the environment (soil, water, sediments, and air).	2001–2010
12	Hylander, L. (6), Meili, M. (5), and Silva, E. (6)	Hg in the environment, particularly in Alto Pantanal; global mercury pollution and expected changes.	2000–2006

Source: Research data. ^1^ Refers to the number of articles published by the author within the corpus.

**Table 2:** Main keywords ranked by frequency in papers dealing with mercury in the Amazon.

Rank	Title and Abstract Words	Frequency
1	Mercury	238
2	Amazon	212
3	Methylmercury	195
4	Fish	147
5	Brazilian Amazon	139
6	Brazil	116
7	Contamination	98
8	Exposure	96
9	Pollution	86
10	Fish consumption	70
11	Hair	64
12	Basin	59
13	Sediments	55
14	Bioaccumulation	42
15	Water	42

Source: Research data.

**Table 3:** Ranking of the top 10 most cited papers within the articles analyzed.

1991–2017					
Rank	Authors (Year)	Cit. ^1^	Co-Cit. ^2^	Journal	Impact Factor (JCR)
1	Malm, O.; Branches, F.; Akagi, H.; Castro, M.B.; Pfeiffer, W.C.; Harada, M.; et al. (1995)	168	80	Science of the Total Environment	4.610
2	Malm, O. (1998)	294	78	Environmental Research	4.732
3	Lebel, J.; Roulet, M.; Mergler, D.; Lucotte, M.; Larribe, F. (1997)	114	75	Water, Air, and Soil Pollution	1.769
4	Roulet, M.; Lucotte, M.; Farella, N.; Serique, G.; Coelho, H.; Passos, C.J.S.; et al. (1999)	138	75	Water, Air, and Soil Pollution	1.769
5	Malm, O.; Pfeiffer, W.C.; Souza, C.M.M.; Reuther, R. (1990)	162	72	AMBIO	3.616
6	Roulet, M.; Lucotte, M.; Saint-Aubin, A.; Tran, S.; Rheault, I.; Farella, N.; et al. (1998)	137	70	Science of the Total Environment	4.610
7	Akagi, H.; Malm, O.; Branches, F.J.P.; Kashima, Y.; Guimaraes, J.R.D.; et al. (1995)	118	68	Water, Air, and Soil Pollution	1.769
8	Fadini, P.S.; Jardim, W.F. (2001)	102	67	Science of the Total Environment	4.610
9	Lebel, J.; Mergler, D.; Lucotte, M.; Amorim, M.; Larribe, F.; Dolbec, J. (1998)	194	62	Environmental Research	4.732
10	Pfeiffer, W.C.; Lacerda, L.D. (1988)	121	60	Environmental Technology Letters	NA
	Total citations	1548	707		

Source: Research data. ^1^ the number of global citations is presented in the column headed Citation; ^2^ the Co-Citation column represents the number of citations received within the 546 articles considered in the analysis, at the moment of the data gathering. JCR: Journal Citation Report. NA: Not available.

## Appendix A

**Top 10 co-cited papers regarding mercury research in the Amazon between 1991 and 2017.**

1. Akagi, H.; Malm, O.; Branches, F.J.P.; Kinjo, Y.; Kashima, Y.; Guimaraes, J.R.D.; Oliveira, R.B.; Haraguchi, K.; Pfeiffer, W.C.; Takizawa, Y.; Kato, H. Human exposure to mercury due to goldmining in the Tapajos River Basin, Amazon, Brazil: Speciation of mercury in human hair, blood and urine. *Water Air Soil Pollut.* **1995**, *80*, 85–94.
2. Fadini, P.S.; Jardim, W.F. Is the Negro River Basin (Amazon) impacted by naturally occurring mercury? *Sci. Total Environ.* **2001**, *275*, 71–82.
3. Lebel, J.; Roulet, M.; Mergler, D.; Lucotte, M.; Larribe, F. Fish diet and mercury exposure in a riparian Amazonian population. *Water Air Soil Pollut.* **1997**, *97*, 31–44.
4. Lebel, J.; Mergler, D.; Lucotte, M.; Amorim, M.; Larribe, F.; Dolbec, J. Neurotoxic effects of low-level methylmercury contamination in the Amazonian Basin. *Environ. Res.* **1998**, *79*, 20–32.
5. Malm, O.; Pfeiffer, W.C.; Souza, C.M.M.; Reuther, R. Mercury pollution due to gold mining in the Madeira River basin, Brazil. *AMBIO* **1990**, *19*, 11–15.
6. Malm, O.; Branches, F.; Akagi, H.; Castro, M.B.; Pfeiffer, W.C.; Harada, M.; Bastosa, W.R.; Kato, H. Mercury and methylmercury in fish and human hair from the Tapajós river basin, Brazil. *Sci. Total Environ.* **1995**, *175*, 145–150.
7. Malm, O. Gold mining as a source of mercury exposure in the Brazilian Amazon. *Environ. Res.* **1998**, *77*, 73–78.
8. Pfeiffer, W.C.; Lacerda, L.D. Mercury inputs into the Amazon Region, Brazil. *Environ. Technol. Lett.* **1988**, *9*, 325–330.
9. Roulet, M.; Lucotte, M.; Farella, N.; Serique, G.; Coelho, H.; Passos, C.J.S.; de Jesus da Silva, E.; Scavone de Andrade, P.; Mergler, D.; Guimarães, J.-R.D.; et al. Effects of recent human colonization on the presence of mercury in Amazonian ecosystems. *Water Air Soil Pollut.* **1999**, *112*, 297–313.
10. Roulet, M.; Lucotte, M.; Saint-Aubin, A.; Tran, S.; Rhéault, I.; Farella, N.; De Jesus Da silva, E.; Dezencourt, J.; Sousa Passos, C.J.; Santos Soares, G.; et al. The geochemistry of mercury in central Amazonian soils developed on the Alter-do-Chão formation of the lower Tapajós River Valley, Pará state, Brazil. *Sci. Total Environ.* **1998**, *223*, 1–24.
